# Postprandial Vascular Effects of a High Potassium Meal in Patients with Treated Hypertension

**DOI:** 10.3390/nu17010045

**Published:** 2024-12-27

**Authors:** Daniela Malta, Sam Esfandiari, Livia A. Goldraich, Johane P. Allard, Gary E. Newton

**Affiliations:** 1 School of Nutrition, Toronto Metropolitan University, 350 Victoria St., Toronto, ON M5B 2K3, Canada; 2 Institute of Medical Science, University of Toronto, 1 King’s College Circle, Toronto, ON M5S 1A8, Canada; esfandiari.sam@gmail.com (S.E.); gary.newton@sinaihealthsystem.ca (G.E.N.); 3 Division of Cardiology, Department of Medicine, Sinai Health System, 600 University Avenue, Suite 427, Toronto, ON M5G 1X5, Canada; 4 Division of Cardiology, Hospital de Clinicas de Porto Alegre, Porto Alegre 90035-903, Brazil; lgoldraich@gmail.com; 5 Department of Nutritional Sciences, University of Toronto, Fitzgerald Building, 150 College Street, Toronto, ON M5S 3E2, Canada; dr.johane.allard@uhn.ca; 6 Division of Gastroenterology, Department of Medicine, Toronto General Hospital, 9N-973, 585 University Avenue, Toronto, ON M5G 2N2, Canada

**Keywords:** potassium, postprandial, clinical nutrition, endothelium, hypertension, dietary intake, flow-mediated dilatation, randomized crossover trial, sodium, vasodilation

## Abstract

Background: There is compelling evidence of an inverse association between potassium intake and blood pressure (BP). A potential mechanism for this effect may be dietary potassium-mediated augmentation of endothelium-dependent relaxation. To date, studies have investigated potassium intake supplementation over several weeks in healthy volunteers with variable results on vascular function. There is no assessment of the acute vascular effects of potassium supplementation achieved by the ingestion of potassium-rich food in a hypertensive population. Objective: The purpose of this study was to investigate the effect of a high potassium meal on postprandial endothelial function as measured by flow-mediated dilatation (FMD). Methods: We performed an investigator-blinded randomized crossover trial in 33 treated hypertensive individuals. Participants consumed both a high (~2400 mg) and low (~543 mg) K^+^ meal, separated by a one-week washout period. The primary endpoint was endothelial function as assessed by FMD pre-meal and postprandially at 60 and 120 min. Meals were compared at each time point using the Hills–Armitage approach. Results: 33 individuals were included in the study (48% male, mean age 68). In the fasting state (Baseline), and at 60 min postprandial, radial artery FMD was not significantly different between the participants after consumption of either meal (baseline: high K^+^ 4.2 ± 2% versus Low K^+^ 2.6 ± 3%, *p* = 0.93; 60 min: high K^+^ 3.8 ± 4% versus Low K^+^ 4.1 ± 3%, *p* = 0.69). However, at 120 min, FMD tended to be higher in participants after the high K^+^ meal (5.2 ± 4.1%) than after the low K^+^ meal (3.9 ± 4.1%) (*p* = 0.07). There were no differences in participants’ radial artery diameter and blood flow between meals. Conclusions: This study does not support our hypothesis that a single high K^+^ meal improves vascular function in individuals with treated hypertension. This does not contradict the clinical evidence relating greater K^+^ intake with lower BP, but suggests that mechanistic investigations of increased K^+^ intake through diet alone and its impact on endothelial function as a mediator to reducing BP are complex and not simply due to single nutrient-mediated improvement in vascular function.

## 1. Introduction

Increasing dietary potassium (K^+^) intake is widely supported and has multiple favorable consequences, including mitigating diabetes risk, improvements in general health, and cardiovascular benefits [1], in particular in the setting of hypertension. Indeed, the Dietary Approaches to Stop Hypertension (DASH) diet, which is high in K^+^, is recommended for the management of hypertension [2]. The blood pressure- (BP) lowering benefits [3] are presumed to be in part due to the high K^+^ nature of the diet [4]. The exact mechanism of how K^+^ intake impacts BP is still unknown [5] but may include direct vascular effects, an autonomic and nitric oxide-mediated effect [6,7,8,9], and indirect effects due to reduced dietary sodium (Na^+^) intake [10], a predictable response to the dietary changes necessary to augment K^+^ intake.

The postprandial state results in multiple cardiovascular adaptations, including an increase in heart rate, stroke volume, and cardiac output; a fall in total peripheral resistance; maintenance of blood pressure; dilation of arterioles in the splanchnic bed; and an increase in intestinal circulation and blood flow. In the periphery, the postprandial vasculature response is highly dependent on meal composition [11]. Postprandially, high fat, glucose, and Na^+^ content in meals are associated with impaired endothelial-dependent vasorelaxation [12,13,14,15], resulting in stiffer arteries [16] and an increased risk of cardiovascular disease [12]. In contrast, both meals and short-term diets rich in antioxidants [13] and K^+^ [17,18], are reported to counter these detrimental effects, at least in part through the release of nitric oxide (NO) [16], a potent vasodilator.

To date, studies assessing K^+^ intake have investigated supplementation over several weeks, have focused on healthy individuals, and have included varying amounts of Na^+^ intake [17,18,19,20]. To our knowledge, there is no assessment of the acute impact of K^+^ supplementation achieved by ingestion of K^+^-rich food on vascular function in a cardiac population. This is an exploratory study to investigate whether acute impact of K^+^ intake on endothelial vasodilation is contrary to the effect of high sodium and high-fat meals and diets which have been both associated with acute impairment of vascular function [14,15], with long-term consequences and cardiac disease progression. In clinical cases where it is crucial to have an impact BP in a short time period, this study might have merit for quick treatment. The rationale for investigating a cardiac population is to explore this question in a clinically relevant setting, and in a cohort of individuals that are likely to have endothelial dysfunction at baseline [21]. The purpose of this investigation was to assess the effect of dietary K^+^ on the endothelium in the postprandial state. We hypothesized that acute intake of a high K^+^ meal would improve postprandial endothelial function compared to a low K^+^ meal.

## 2. Subjects and Methods

We performed an investigator-blinded randomized crossover trial. Study participants were recruited through a general cardiology outpatient clinic. The diagnosis of hypertension was confirmed by the participants’ cardiologists. Recruitment also occurred via an online community and by a flyer available in the clinic area. If recruited from the online community or flyer, hypertension was self-reported and confirmed by study investigators based on a participant’s medical prescriptions. Investigators blinded to data acquisition and analysis performed meal allocation by using excel software, selecting, at random, the first meal consumed with varying order one week later. Participants consumed both a high (~2400 mg of K^+^) and low (~543 mg of K^+^) K^+^ meal on different study visits, separated by a one-week washout period. The primary endpoint was endothelial function as assessed by flow-mediated dilatation (FMD). Additional exploratory outcomes included serum K^+^, Na^+^, glucose, triglycerides, heart rate, and systolic and diastolic blood pressure.

Participants were included if they had controlled (treated) hypertension, were 18–80 years of age and had normal sinus rhythm. Participants were excluded if they had a recent hospital admission due to a cardiovascular event, had a serum K^+^ concentration >5.0 mmol/L, currently smoked, were pregnant, were receiving insulin therapy, had a glomerular filtration rate <90 mLs/min/1.73 m^2^, were on loop diuretic therapy, or had any food allergies or intolerance to meal items. This study was approved by the Research Ethics Board at Mount Sinai Hospital in Toronto, Canada. All patients provided written informed consent for their participation.

### 2.1. Study Visits

Participants came to the hospital for two study visits in the fasted state. If participants were recruited from an online community or via posters, they were required to attend a screening visit to ensure eligibility. During the screening visit, past medical history was documented as well as height, weight, and 5 ambulatory BPs, heart rate, and medications. Participants recruited from the clinic were screened via their patient chart. Both study visits occurred at the Cardiovascular Clinical Research Laboratory at Mount Sinai Hospital after a fasting period of 8 h. Medications were held the morning of study days. Patients also abstained from caffeine and exercise on each study day and were instructed to eat similar meals the day before each study visit. To ensure diet was similar before each visit, participants were required to complete a 3-day food record before each study visit and were given a copy of the last day of the food record (the day previous to the visit) to aim to consume similar foods prior to the second visit. The nutrient content of foods and beverages was quantified with a specialized software package for dietary analysis (ESHA Food Processor SQL Version 10.14.1, ESHA Research Inc., (Salem, OR, USA)). Participants were not instructed to modify their diet in any way before and during the study.

Anthropometrics were taken when the participant first arrived for the study visit; thereafter, participants were instructed to rest in a supine position in a quiet room for 20 min. Brachial BP readings were then taken using a calibrated sphygmomanometer. A mean of 5 BP measurements was used. A 5F cannula was inserted percutaneously into the antecubital vein of the non-dominant arm under local topical anesthesia (lidocaine 1%) to ensure easy blood draws at various time points throughout the visit (pre-meal and 60 and 120 min postprandial). Biochemistry was drawn for assessment (pre-meal and 60 and 120 min postprandial) of acute changes in serum electrolytes and lipids in response to meal ingestion. Baseline assessment of endothelial function was captured using FMD. After completion of baseline FMD, patients were randomized to receive either the high or low K^+^ meal. Periods 1 and 2 refer to the first and second meal tests, consumed in random order (either high K^+^ or Low K^+^ meals).

Patients consumed the meal over a 20-min period. After consumption, the study investigator retrieved the meal and documented any leftovers. Blood pressure, biochemistry, and FMD were repeated in the postprandial state at 60 and 120 min. Participants returned to the hospital one week later to receive the other intervention. The same sequence of methods was applied. All study visits and data acquisition were completed at Mount Sinai Hospital, Toronto, Canada, in the cardiovascular research laboratory.

### 2.2. Meal Compositions and Considerations

All meals were prepared in the same prep area using preparation methods that were determined a-priori. Meals were designed for a breakfast meal. A combination of foods was selected to ensure intakes were isocaloric and had similar quantities of Na^+^, total fat, saturated fat and total carbohydrates. In the designed meals, only K^+^ intake was planned to be drastically different (~2395 mg vs. ~543 mg of K^+^). In addition, all meals were controlled for temperature and all food products were purchased at the same grocery store by study investigators to minimize the variation in the K^+^ content of foods. Nutrient values were calculated based on data provided from the Canadian Nutrient File [22] and from the products nutrient facts tables. Foods items were not analyzed directly for nutrient content. Potassium was not added to the meals. Potassium content of meals was derived from inherent K^+^ content of foods consumed.

### 2.3. Assessment of Endothelial Function

Flow-mediated dilatation is a non-invasive, endothelial-dependent index of endothelial function. Flow-mediated dilatation is an indirect measure of NO [23], a major mediator in vasodilation [24]. In this method, ultrasound is used to measure the caliber of the radial artery in response to an acute increase in blood flow. Ultrasound images and continuous videos were obtained with a 14 MHz linear array transducer and a commercially available platform (Vivid7, GE Healthcare; Horne, Norway). The increase in blood flow is achieved by inflating a BP cuff distal to the ultrasound probe to a level above the patient’s systolic BP to reduce local blood flow. The cuff release results in a sudden increase in blood flow (reactive hyperemia), causing endothelium-dependent dilatation of the conduit artery, which is measured with a stationary ultrasound probe. All endothelial function parameters are obtained in a supine position in a quiet, temperature- and humidity-controlled room. The baseline radial artery diameter is averaged from 3 images taken at 5-s intervals. After a 5-min recording of resting radial artery diameter and blood flow measurement, the pneumatic cuff (at the wrist distal to the site of radial artery measurement) is inflated to 250 mmHg for 4.5 min. Upon release of the cuff, blood flow under the transducer suddenly increases due to maximal vasodilation of the territories exposed to ischemia. Flow- mediated dilatation measurement starts immediately after cuff deflation and is calculated as the percent change in vasodilation comparing the radial artery maximal diameter at 4.5 min after cuff deflation and the radial artery diameter at baseline. Blinded analysis was performed using a custom-designed automatic vascular edge detection software. Radial artery diameter was calculated using a semi-automatic computerized system that allows continuous measurement of the arterial diameter through resting conditions, cuff inflation, and after cuff deflation.

## 3. Statistical Analysis

Sample size was calculated based on our primary endpoint, FMD. Sample size estimates were considered using data in which hypertensive volunteers had a significantly lower FMD (3.6 ± 2.8%) compared to normotensive middle-aged volunteers (6.1 ± 4.3%) [25]. Arbitrarily, we set a 50% change in FMD to be meaningful. To detect this difference, with 80% power, a sample size of 30 patients was needed.

Continuous variables are described as means and standard deviations. Categorical variables are described as frequencies and percentages. Between-period baseline comparisons were analyzed using an independent sample *t*-test for continuous variables and a chi-square test was used for categorical variables, or where necessary, Fisher’s exact test was used. Meals were compared at each time point postprandially (60 and 120 min) using the Hills–Armitage approach [26] (ANOVA-based) for crossover trials, which uses independent sample *t*-tests to make inferences about differences of interest in crossover studies, such as period and treatment differences. For primary and secondary outcomes, a pooled treatment analysis with the assumption of equal variances, reporting differences between treatments at respective time points is discussed. The pre-meal time point was not included in the Hills–Armitage analysis; however, an additional baseline (pre-meal) between-meal comparison was performed using an independent sample *t*-test. All statistical analyses were performed using SAS version 9.4 and IBM SPSS version 20. An alpha of *p* < 0.05 was considered significant.

## 4. Results

Thirty-eight individuals were randomized. The intervention was discontinued for three individuals due to their inability to consume the study meal on account of diabetes diagnosis and non-compliance of the study protocol. One individual was lost to follow-up. Thirty-three participants completed the study with all outcome data obtained. A flowchart outlining the study design is provided in Appendix A. Baseline characteristics are presented in Table 1. There were no statistically significant differences in age, sex, body mass index, BP, and heart rate between participants in period 1. Medication use and comorbidities were similar between participants in period 1. Intake of nutrients consumed by study participants is presented in Table 2. Caloric, fat, and saturated fat intake were similar between meals. Potassium, Na^+^, and all carbohydrates were statistically different between meals. Potassium intake was 300% higher in the high K^+^ meal compared to the low K^+^ meal.

### 4.1. Endothelial Function—Radial Flow-Mediated Dilatation

**Figure 1:**
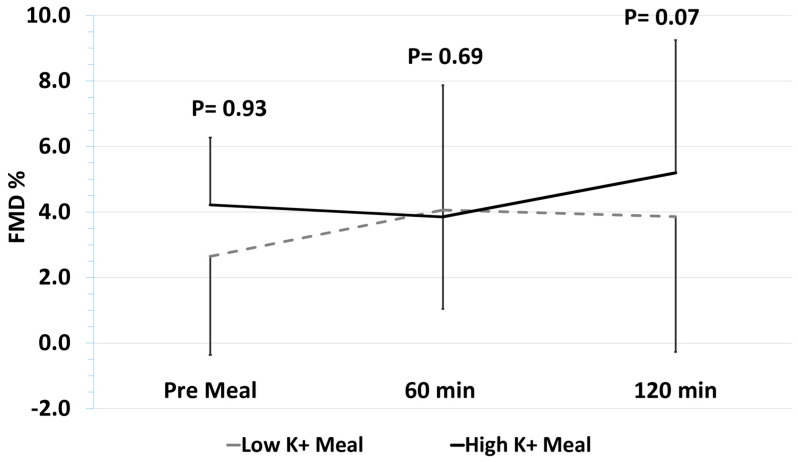
Radial artery flow-mediated dilatation original to this manuscript. Data are mean ± standard deviation. *n* = 33, 184 FMD observations used. For 60 and 120 min time points, *p* represents the Hills–Armitage approach (ANOVA-based) comparing each respective treatment timepoint. Reported is a pooled analysis with assumptions of equal variances, reporting differences between treatments. An additional baseline between-meal comparison was performed using an independent sample t-test (*p* = 0.93). Dashed line represents the low potassium meal; solid line represents the high potassium meal. FMD = Flow-Mediated Dilatation.

In the fasting state (baseline), and at 60-min postprandial, the participants’ radial artery FMD was not significantly different between intake of high and low K^+^ meals (baseline: high K^+^ 4.2 ± 2% versus low K^+^ 2.6 ± 3%, *p* = 0.93; 60 min: high K^+^ 3.8 ± 4% versus low K^+^ 4.1 ± 3%, *p* = 0.69) (Figure 1). At 120 min, FMD tended to be higher in participants after the high K^+^ meal (5.2 ± 4.1%) than after the low K^+^ meal (3.9 ± 4.1%) (*p* = 0.07). There were no differences between participants when consuming low and high K^+^ meals in radial artery diameter, and radial artery blood flow, represented as % change from baseline to post cuff deflation, and reactive hyperemia (Table 3). An additional crossover analysis of FMD showed no significant interaction of diet and time between meals (*p* = 0.62), further confirming the above-mentioned results.

### 4.2. Blood Pressure, Serum Biochemistry and Dietary Intake

Consistent with the intervention, serum K^+^ concentrations were significantly increased in participants at 60 and 120 min following the high K^+^ meal when compared with the low K^+^ meal (Table 4). Sodium concentrations remained similar between participants despite meals. Comparing participants’ results following the Low K^+^ meal to High K^+^ meal at each time point, serum glucose was significantly higher after the Low K^+^ meal at 60 min (*p* = 0.005) but not at 120 min; serum triglycerides were significantly higher after the High K^+^ meal at 60 min (*p* = 0.03) but not at 120 min. There were no observed differences between participants despite meals consumed for serum protein, HDL, LDL and insulin. Details of all biochemical responses are summarized in Table 4. There were minor blood pressure changes at 120 min, only achieving significance for diastolic BP, with no changes in HR in participants between consumption of the two meals (Table 4). Dietary intake before both the low K^+^ and high K^+^ meals are reported in Table 5. There were no differences in the 3-day reported dietary intake before each study meal.

## 5. Discussion

The purpose of this investigation was to understand the acute effects of K^+^ intake on endothelial function after a complete breakfast meal in hypertensive individuals. Contrary to our hypothesis, our study demonstrated that despite a significant increase of K^+^ ingested in a single meal, endothelial function, as measured by FMD, was not significantly altered.

A meta-analysis published in 2017 [27] summarized the effects of K^+^ supplementation (dosage ranging from 40 to 150 mmol/day, and intervention ranging from 6 days to 12 months) on markers of vascular function. Supplementation had no effects on pulse wave velocity and FMD. The 3 FMD trials included in this meta-analysis reported variable FMD results. Blanch et al., who investigated high K^+^ diet (150 mmol/day) in normotensives [18] and, He et al. [28], who investigated potassium chloride supplementation (64 mmol daily) in hypertensives, showed improved FMD with K^+^ supplementation. In contrast, Berry et al. provided the least amount of K^+^ (40 mmol/day) to participants through K^+^ citrate supplementation, investigated individuals with early HTN, and reported no benefit of K^+^ supplementation on FMD [29]. The most recent meta-analysis evaluating the effect of K^+^ intake, whether from dietary sources or supplementation, on flow-mediated dilation (FMD) demonstrated that K^+^ supplementation is associated with a significant improvement in endothelial function [30]. Specifically, Gijsbers et al. reported an increase in FMD of 1.6% following K^+^ chloride supplementation (2.8 g/day for 4 weeks) [20]. In addition, Smiljanec et al. found that K⁺ supplementation mitigated the negative vascular effects associated with high sodium intake [31]. These studies predominantly included healthy individuals or participants with borderline hypertension who were not on antihypertensive medications. Only two additional trials were incorporated into this meta-analysis compared to the earlier analysis by Tang et al. [27], further reinforcing the vascular benefits of K^+^ supplementation. A trial investigating the impact of the DASH diet on vascular function reported no significant alterations in FMD after a 30-day intervention despite significant reductions in BP [32]. The study duration ranged from 6 days to 6 weeks. An acute study investigating the postprandial effects of K^+^ supplementation in normotensive participants by Blanch et al. [17], reported that a high K^+^ meal (36 mmol, 1400 mg K^+^) significantly attenuates the postprandial decrease in FMD compared to a low K^+^ meal. This group went on to investigate the effects of adding varying amounts of Na^+^ to high (38 mmol, 1482 mg K^+^) and low (3 mmol, 117 mg K^+^) K^+^ meals [19]; in both diets, the addition of K^+^ attenuated postprandial decreases in FMD, regardless of Na^+^ content. Methodological differences exist in our study from investigations by Blanch et al. [17] and may explain the contrast in findings. We provided participants with a much higher amount of K^+^ (59 mmol, 2301 mg K^+^). Further, the quantity of food provided in our meals was larger and we provided participants with complete meals as opposed to one food item. Our participants were hypertensive and receiving medical therapy. These considerations can impact endothelium performance and subsequently postprandial vasodilation. To our knowledge, placing the current study in the context of the larger body of literature is difficult as there is a lack of literature investigating potassium’s effects on endothelial function in the postprandial state. Blanch et al. [17] provide one of the most similar insights in technique and methodologies to the current study.

The postprandial FMD response in hypertensive individuals is unknown, as studies investigating FMD in the postprandial state have focused largely on normotensive individuals [15,17,19]. One study in hypertensive males investigated the postprandial FMD response of 2 meals (2 slices of white bread with sesame oil or either corn or olive oil). Investigators reported increases in FMD after consumption of meals containing sesame oil but not with the other oils [33]. To our knowledge, our study is the first to investigate the postprandial effects of a K^+^ dense meal. Even in health, there is no clear consensus on what a normal postprandial FMD response is. Theoretically, it is thought that FMD should decrease in the periphery post meal, although studies investigating varying meal compositions report both increases [34], decreases [13,15,17,35,36], and neutral effects [13]. This highlights the challenges in understanding the postprandial response related to meal composition and vascular function in health and disease.

Several characteristics of our study design merit discussion. We conducted a randomized crossover trial in order to reduce variability. Given the short duration of the intervention and a washout period of 1 week, there is no evidence of carryover or treatment effect. Important to acquiring reliable FMD results is having a controlled, temperature-regulated environment. We have used the same laboratory, protocols, and temperature conditions for FMD studies over the last decade [25,37,38] and previously reported the repeatability (range of variation = 1.7% (0.1% to 5.2%; coefficient of repeatability = 3.2%) of this technique in our setting [37]. The current study is relatively small in nature; however, a sample size calculation was conducted to ensure adequate statistical power. While we acknowledge the potential for errors, such as inaccuracies in sample size estimation or variance assumptions, we have taken measures to minimize these risks. Specifically, the estimates used for the sample size calculation were derived from data generated in our laboratory using a population similar to the current study [25]. Study meals and meal preparation protocols were designed by a registered dietitian and matched for energy, Na^+^ and fat. This is of particular importance as all these factors can confound the results of our primary endpoint [14,15,39] and have been shown to affect postprandial vascular function.

A particular strength of this study was the quantity of K^+^ provided (~2400 mg) in a complete meal (300% more K^+^ than the low K^+^ meal). This is approximately half of the total daily adequate intake recommended to adults by the Institute of Medicine [40]. This increase in K^+^ was supported by the significant differences observed in serum K^+^ concentrations between time points. Although there were slight differences in other nutrients provided between interventions, K^+^ intake drastically exceeded any differences in other nutrients. Of note, we measured actual intake, in contrast to other investigations, which either did not measure this quantity, or provided single food source meals. A priori controls were implemented to minimize potential variability and confounding effects of food intake and dietary patterns prior to the trail days. Participants were instructed to replicate their dietary intake from the day prior to each study visit, supported by the completion of a 3-day food record. The record from the final day was provided to participants as a reference to enhance consistency in pre-visit dietary patterns. Our results indicated that a participant’s dietary intake prior to each study visit was similar (Table 4). During study visits, dietary intake was standardized and closely monitored. Meals were prepared consistently by the same investigator using uniform methods across visits, with uneaten food recorded and included in intake calculations. Participants were also required to fast and withhold medications on the morning of each visit. While these measures taken reduced variability, some degree of variation is unavoidable in human studies and is acknowledged. Finally, this is the first study to investigate the acute effects of K^+^ intake in stable, medicated, hypertensives, a population likely to have endothelial dysfunction at baseline.

Some limitations to our study warrant discussion. Investigating endothelial function in individuals who have endothelial damage at baseline is complex, with many factors that may confound or alter the endothelium’s ability to respond to meal ingestion. Endothelial dysfunction is a prelude to the development of hypertension [21] and thus many individuals living with hypertension have some degree of dysfunction [41]. Forty-five percent of participants were former smokers, a factor that may have influenced baseline endothelial dysfunction. Smoking is a well-documented contributor to endothelial impairment through mechanisms such as increased oxidative stress, reduced nitric oxide bioavailability, and vascular inflammation [42]. Despite its relevance, the current study did not assess the cumulative smoking burden (e.g., pack–years) or the time elapsed since smoking cessation, both of which are critical determinants of vascular recovery and endothelial function restoration. In addition, age-related declines in endothelial function have also been extensively documented in the literature [43]. This observation may suggest the presence of baseline endothelial dysfunction within the current study population. Potassium’s theoretical ability to acutely mitigate the damaged endothelium to increase vasodilatory properties is unknown. Furthermore, our study did not provide evidence in support of this concept.

Postprandial hyperglycemia and hyperlipidemia are known causes of endothelial dysfunction in the postprandial state [34] and are identified as contributors to prolonging the post-absorptive state. We showed significant increases in both glucose and triglycerides at 60 min, thereafter triglycerides continuing to increase at 120 min while glucose tapering down, coincident with a trend towards significance in FMD. Fats and cholesterol remained constant (values not reported here). In addition, despite efforts to try to match for carbohydrates in the present study, total carbohydrates and sugar intake were higher in the low K^+^ meal than the high K^+^ meal. Overall fiber intake was lower in the low K^+^ meal than in the high K^+^ meal. This is an important factor to consider, as high fiber intakes may affect digestive transit time and thus affect the rate of absorption. It has been reported that the type of carbohydrate intake (i.e., a high-glucose meal versus a high-fiber meal) affects the endothelium differently and should be considered when interpreting results following a complex dietary intervention. Lavi et al. [44] reported that ingestion of a high-glucose meal suppressed postprandial FMD (compared to baseline) while no difference was observed for the high-fiber meal. In other acute studies, FMD was suppressed following an oral glucose load [39,45].

In our study, participants were receiving various classes of antihypertensive medications. Potassium’s role in enhancing the efficacy of antihypertensive medications is well documented. For instance, K^+^ supplementation has been shown to reduce the occurrence of hypokalemia in patients using thiazide diuretics [46], a common side effect due to increased renal K^+^ excretion. Additionally, ACEi, another commonly prescribed class of antihypertensive drugs, influence drug metabolism and renal handling of K^+^ [47]. As hyperkalemia is a well-documented potential side effect of ACEi initiation, careful monitoring of K^+^ intake and serum levels is essential during initial treatment to mitigate associated risks and optimize therapeutic outcomes. Notably, ACEi have been associated with improvements in endothelial function, particularly in the postprandial state [48], a finding relevant to our investigation. However, the effects of other antihypertensive medications on endothelial function remain less understood. To account for potential variability and confounding effects, all participants in our study were on stable antihypertensive regimens for three months prior to participation. Furthermore, to minimize acute influences of these medications on study endpoints, participants were instructed to withhold their medications on the morning of each trial visit. These precautions were essential to isolating the specific effects of K^+^ interventions while acknowledging the complex interplay between antihypertensive therapies and vascular function.

Lastly, the designed meals were not sent to a food lab for analysis of K^+^ content. Variations were not expected in the designed meals regarding the K^+^ content as the values were calculated using Canadian Nutrient File and Nutrition Facts Labels. Also, the food products were purchased by the same investigator from the same grocery store for the duration of the study.

## 6. Conclusions

The findings of this study add to the current body of literature in this small and specified area of postprandial research involving dietary intake and endothelial function. However, the results do not support the hypothesis that the consumption of a single high- K^+^ meal improves vascular function, as assessed by FMD, in individuals with treated hypertension. There are many factors to consider when interpreting the outcomes of this study. The results may suggest that the beneficial effects of dietary K^+^ on vascular health may not manifest acutely, potentially requiring sustained dietary modifications or consistent supplementation to observe measurable improvements in endothelial function. Alternatively, the lack of effect might indicate that altering a single nutrient, such as K^+^, in isolation is insufficient to influence FMD, particularly in a population with established hypertension, ongoing treatment regimens, and potential endothelial dysfunction at baseline. Furthermore, the inherent complexity of whole foods may introduce unknown dietary interactions that attenuate the K^+^ effect on endothelial health.

The results underscore the complex interplay between dietary interventions and vascular function, highlighting the need for studies to explore dietary K^+^ intake, its integration with other dietary components, and its cumulative impact on endothelial health. Additionally, the potential role of individual variability, medication interactions, and baseline endothelial dysfunction may further moderate the observed effects, warranting further investigation into tailored dietary strategies for optimizing future study design and methodologies.

## Figures and Tables

**Table 1:** Baseline characteristics (Pre-meal).

	Total *n* = 33	High K^+^ Meal First *n* = 15	Low K^+^ Meal First *n* = 18	*p*
Age (y)	63 ± 10	64 ± 10	62 ± 10	0.63
Men (%)	16 (48)	5 (33)	11 (61)	0.17
BMI (kg/m^2^)	27 ± 4	28.5 ± 4	27 ± 5	0.47
Systolic BP (mmHg)	124 ± 11	128 ± 12	122 ± 8	0.09
Diastolic BP (mmHg)	72 ± 8	72 ± 9	71 ± 7	0.71
Heart Rate (bpm)	64 ± 10	63 ± 9	64 ± 11	0.87
Diabetes (%)	1 (3)	0	1 (5)	1.00
Smoking Hx (%)	15 (45)	6 (40)	9 (50)	0.74
Calcium channel blocker (%)	15 (45)	6 (40)	9 (50)	0.73
Thiazide Diuretic (%)	10 (30)	4 (27)	6 (33)	0.54
ACE inhibitor/ARB (%)	27 (82)	12 (80)	15 (83)	1.00
Beta Blocker (%)	4 (12)	2 (13)	2 (11)	1.00
Statin (%)	13 (39)	6 (40)	7 (39)	1.00
Vitamin/mineral use (%)	18 (55)	10 (67)	9	0.48

*p* < 0.05 is considered significant. Independent sample *t*-test was used for continuous variables; Chi-square test was used for categorical variables, or where necessary, Fisher’s exact test was used. Data are mean ± SE for continuous variables. Categorical variables are described as frequencies ± percentages. Dashes in the *p* column indicated non-significance.

**Table 2:** Pre-determined nutrient targets and actual nutrient intake.

*n* = 33	High K^+^ Meal		Low K^+^ Meal	
Nutrients	Nutrient Target	Actual Nutrient Intake	Nutrient Target	Actual Nutrient Intake
Energy, kcal	701	676 ± 41	691	672 ± 43
Total Fat, g	7.0	6.8 ± 0.5	6.89	6.4 ± 1.6
Saturated, g	3.5	3.3 ± 1	3.5	3.2 ± 1
Total Carbohydrates, g	136.0	131 ± 8	156.0	151 ± 11 *
Sugar, g	85.8	82 ± 6	94.0	90 ± 10
Fiber, g	16.0	15.0 ± 1	7.9	8 ± 0.5 *
Protein, g				
Sodium, mg	571	557 ± 30	501	476 ± 83 *
Potassium, mg	2395	2278 ± 183	543	530 ± 35 *

Data are: Nutrient target reflects a-priori nutrient target of the designed meal (total amount). Actual nutrient intake reflects actual nutrients consumed by study participants during study visits. Actual intakes: values are mean ± SD. *p* ≤ 0.05, * represents significant difference using a paired sample *t*-test; means compared between actual intake variables (Low K^+^ vs. High K^+^ meal intake).

**Table 3:** Radial artery diameter and reactive hyperemia.

*n* = 33	Pre Meal		60 min Post Meal		120 min Post Meal	
Meal	Radial Artery, mm	Reactive Hyperemia, %	Radial Artery, mm	Reactive Hyperemia, %	Radial Artery, mm	Reactive Hyperemia, %
Low K^+^	0.27± 0.06	620.8 ± 628.7	0.27 ± 0.06	646.1 ± 494.8	0.26 ± 0.06	673.8 ± 454.7
High K^+^	0.27 ± 0.06	710.8 ± 673.7	0.27 ± 0.06	557.0 ± 502.9	0.26 ± 0.05	503.1 ± 462.1

Data are mean ± standard deviation. The data represents the Hills–Armitage approach (ANOVA-based) which uses two samples *t*-tests for continuous variables. Reported is a pooled treatment analysis with assumption of equal variances, reporting differences between treatments at each respective time point. Baseline between-meal comparisons were performed with an independent sample *t*-test. Reactive hyperemia was calculated as % change of blood flow from baseline to blood flow after cuff deflation.

**Table 4:** Serum biochemistry and hemodynamics.

*n* = 33	Low K⁺ Meal			High K⁺ Meal		
	0 min	60 min	120 min	0 min	60 min	120 min
Serum K⁺ (mmol/L)	3.83 ± 0.39	3.59 ± 0.41	3.62 ± 0.37	3.78 ± 0.46	3.68 ± 0.38 *	3.90 ± 0.52 *
Serum Na⁺ (mmol/L)	142.3 ± 2.5	141.8 ± 2.4	141.7 ± 2.7	141.5 ± 1.8	141.8 ± 2.4	141.9 ± 2.5
Serum Glucose (mmol/L)	5.76 ± 1.10	8.08 ± 3.14	6.46 ± 2.44	5.72 ± 0.92	7.28 ± 2.70 *	6.19 ± 2.02
Serum Triglycerides (mmol/L)	1.13 ± 0.50	1.18 ± 0.50	1.29 ± 0.57	1.27 ± 0.63	1.39 ± 0.77 *	1.48 ± 0.86
Systolic BP (mmHg)	123.3 ± 9.1	124.8 ± 9.9	123.5 ± 10.5	124.3 ±11.1	125.4 ± 10.8	125.8 ± 10.2
Diastolic BP (mmHg)	70.7 ± 8.0	67.8 ± 7.2	68.9 ± 7.9	71.7 ± 8.3	69.2 ± 7.6	70.8 ± 8.7 *
Heart rate (bpm)	62.9 ± 9.5	68.9 ± 12.1	65.8 ± 11.2	62.8 ± 9.7	66.4 ± 9.8	64.2 ± 9.8

Data are mean ± standard deviation. *p* represents the Hills–Armitage approach (ANOVA-based) which uses independent sample *t*-tests. Reported is a pooled analysis with assumptions of equal variances, reporting differences between treatments. Baseline between-meal comparisons were performed with an independent sample *t*-test. * *p* ≤ 0.05 comparing Low K^+^ meal to High K^+^ meal at each time-point. Conversions; 39.10 mg = 1 mmol of K⁺; 23 mg = 1 mmol of Na⁺.

**Table 5:** 3-day reported dietary intake prior to each meal original to this manuscript.

*n* = 33	Low K^+^ Meal	High K^+^ Meal	*p*
Energy, kcal	1862 ± 552	1883 ± 558	0.88
Potassium, mg/day	3266 ± 974	3172 ± 911	0.69
Sodium, mg/day	2282 ± 730	2293 ± 722	0.95
Fat, mg/day	70 ± 29	73 ± 28	0.69
Carbohydrates, mg/day Sugars, mg/day Fiber, mg/day	220 ± 81 86 ± 46 22 ± 8	220 ± 81 89.9 ± 44 21 ± 7	0.97 0.83 0.59

Data are mean ± standard deviation. *p* represents independent sample *t*-test. Note: *p* < 0.05 comparing Low K^+^ meal to High K^+^ meal.

**Figure A1:**
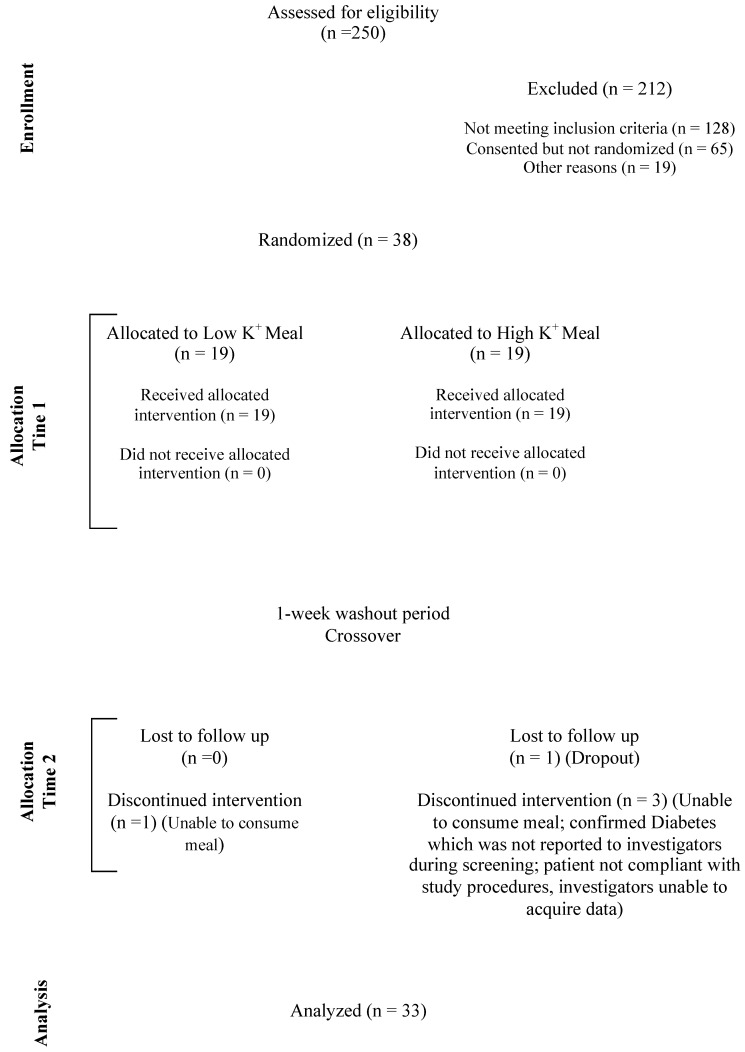
Flow diagram modified from Consort flow diagram for parallel randomized control trials.

## Data Availability

The datasets presented in this article are not readily available because of privacy restrictions.

## Abbreviations

The following abbreviations are use in this manuscript:

K⁺	Potassium
DASH	Dietary Approaches to Stop Hypertension
Na^+^	Sodium
FMD	Flow-Mediated Dilatation
BP	Blood Pressure
